# 3D SASHA myocardial T1 mapping with high accuracy and improved precision

**DOI:** 10.1007/s10334-018-0703-y

**Published:** 2018-09-06

**Authors:** Giovanna Nordio, Aurélien Bustin, Markus Henningsson, Imran Rashid, Amedeo Chiribiri, Tevfik Ismail, Freddy Odille, Claudia Prieto, René Michael Botnar

**Affiliations:** 1 School of Biomedical Engineering and Imaging Sciences, King’s College London, 4th Floor, Lambeth Wing, St Thomas’ Hospital, London, SE1 7EH UK; 2 Escuela de Ingeniería, Pontificia Universidad Católica de Chile, Santiago, Chile; 3 Imagerie Adaptive Diagnostique et Interventionnelle, INSERM et Université de Lorraine, Nancy, France; 4 CIC-IT 1433, INSERM, Université de Lorraine, CHRU de Nancy, Nancy, France

**Keywords:** Myocardial T1 mapping, Precision, Accuracy, Denoising, Cardiac MRI

## Abstract

**Purpose:**

To improve the precision of a free-breathing 3D saturation-recovery-based myocardial T1 mapping sequence using a post-processing 3D denoising technique.

**Methods:**

A T1 phantom and 15 healthy subjects were scanned on a 1.5 T MRI scanner using 3D saturation-recovery single-shot acquisition (SASHA) for myocardial T1 mapping. A 3D denoising technique was applied to the native T1-weighted images before pixel-wise T1 fitting. The denoising technique imposes edge-preserving regularity and exploits the co-occurrence of 3D spatial gradients in the native T1-weighted images by incorporating a multi-contrast Beltrami regularization. Additionally, 2D modified Look-Locker inversion recovery (MOLLI) acquisitions were performed for comparison purposes. Accuracy and precision were measured in the myocardial septum of 2D MOLLI and 3D SASHA T1 maps and then compared. Furthermore, the accuracy and precision of the proposed approach were evaluated in a standardized phantom in comparison to an inversion-recovery spin-echo sequence (IRSE).

**Results:**

For the phantom study, Bland–Altman plots showed good agreement in terms of accuracy between IRSE and 3D SASHA, both on non-denoised and denoised T1 maps (mean difference −1.4 ± 18.9 ms and −4.4 ± 21.2 ms, respectively), while 2D MOLLI generally underestimated the T1 values (69.4 ± 48.4 ms). For the in vivo study, there was a statistical difference between the precision measured on 2D MOLLI and on non-denoised 3D SASHA T1 maps (*P* = 0.005), while there was no statistical difference after denoising (*P* = 0.95).

**Conclusion:**

The precision of 3D SASHA myocardial T1 mapping was substantially improved using a 3D Beltrami regularization based denoising technique and was similar to that of 2D MOLLI T1 mapping, while preserving the higher accuracy and whole-heart coverage of 3D SASHA.

**Electronic supplementary material:**

The online version of this article (10.1007/s10334-018-0703-y) contains supplementary material, which is available to authorized users.

## Introduction

Quantitative myocardial T1 mapping has been explored as a tool to evaluate different cardiomyopathies, particularly to assess myocardial fibrosis [1].

Several T1 mapping imaging techniques have been proposed which use inversion recovery [2, 3], saturation recovery [4, 5] or a combination of both pre-pulses [6]. The majority of these sequences are employed as two-dimensional (2D) imaging techniques where a single 2D map is acquired per breath-hold. However, breath-holding limits the achievable spatial resolution or may introduce slice mis-registration artifacts, which can hinder fibrosis detection in the right ventricle, atrial wall or apex of the left ventricle. A three-dimensional (3D) T1 mapping technique would thus be preferable, as they permit volumetric coverage of the heart in free-breathing with higher signal-to-noise ratio (SNR) and improved image resolution.

Recently, a 3D saturation-recovery-based T1 mapping imaging technique, called 3D SASHA, has been proposed, which permits the acquisition of whole-heart images under free-breathing conditions [7]. This technique has been successfully validated in healthy subjects and showed higher accuracy in the estimation of myocardial T1 than the 2D modified Look-Locker inversion recovery-based (MOLLI) technique used clinically. Although the precision of the 3D SASHA T1 map has been shown to be superior to 2D SASHA, it is still lower than that of 2D MOLLI. This lower precision is due in part to the low dynamic range of signal intensity when using saturation pulses, which makes saturation-recovery-based T1 mapping sequences more sensitive to noise. A precise estimation of myocardial T1 values is important for achieving higher reproducibility and ultimately improving diagnostic utility.

To address this issue, efficient 2D denoising techniques have recently been proposed to reduce noise in the native T1-weighted images while preserving the inherent cardiac structures [8, 9]. Jang et al. [9] employed 2D denoising based on a tissue similarity distance to reduce the sensitivity to noise of 2D STONE [10] T1 mapping when three-parameter fitting is employed. Bustin et al. [8] developed a 2D denoising method using the Beltrami regularization to improve the precision of 2D breath-hold saturation-based myocardial T1 mapping sequences while maintaining accuracy of T1 quantification. This feature-preserving regularization technique can greatly reduce the staircase effect [11] often associated with other regularity priors. In this study, we investigated the use of the Beltrami regularization denoising approach to further improve the precision and image quality of the 3D free-breathing SASHA sequence. The Beltrami denoising framework will be investigated to exploit the spatio-contrast variation correlation in the native 3D T1-weighted images.

## Materials and methods

### 3D denoising

The Beltrami regularization for image denoising and enhancement was introduced for 2D natural images by Sochen et al. [12] and was proposed for 2D MRI myocardial T1 mapping denoising by Bustin et al. [8]. The potential of the Beltrami regularization framework lies in the general definition of the space-feature manifold and the choice of its metric. In particular, the regularization can be chosen such that the energy of the unknown image corresponds to an arbitrary interpolation between quadratic or total variation gradient penalties.

In this work, we apply the Beltrami denoising framework to 3D T1 mapping by exploiting the 3D spatial and T1 recovery dimension redundant information. This approach thus exploits the 3D spatial gradients of each T1-weighted image and the common edge information between T1-weighted images with varying contrast. A coupling between the T1-weighted images is performed as described by Bustin et al. [8], to enforce common edge information across the 3D images in the T1 encoding direction. With this approach, common structures/edges in different T1-weighted images are preserved, while local intensity variations specific to each T1-weighted image are treated as noise, and thus reduced. Therefore, edges/details in the images will be better enhanced if they are present in multiple T1-weighted images.

Following the derivation in Bustin et al. [8], the 3D Beltrami denoising framework for 3D T1 mapping can be expressed as the following optimization problem:

$$\hat{m}_{i} = {\text {argmin}}_{{m_{i} }} \left\{ { \| m_{i} - d_{i} \|_{2}^{2} + \lambda \sqrt {1 + \beta^{2} \mathop \sum \limits_{i = 1}^{n} \left| {\nabla^{w} m_{i} } \right|^{2} } } \right\},$$

where $\{ \hat{m}_{i} \}_{i = 1}^{n}$ are the denoised 3D T1-weighted images for the contrasts $i = 1, \ldots ,n$, $\{ d_{i} \}_{i = 1}^{n}$ are the corresponding 3D T1-weighted images before denoising, $\lambda$ is the regularization parameter that controls the trade-off between the fidelity to the original acquired data and the Beltrami regularization term, $\beta$ is the Beltrami constant that allows selection of any arbitrary interpolation between quadratic or total variation gradient penalties, and $\{ \nabla^{\text{w}} m_{i} \}_{i = 1}^{n}$ are the 3D weighted-gradient transformation applied to the 3D T1-weighted images with varying contrast. The weighted-gradient functions penalize the gradients depending on their local orientation in order to reduce local smoothing in the directions where sharp transitions are observed, with $w(m_{i} ) = e^{{ - \frac{{(\nabla m_{i} )^{2} }}{{h^{2} }}}}$ updated at each iteration of the optimization and $h$ as a smoothing parameter.

The optimization problem described above was solved using an efficient and simple primal–dual hybrid gradient algorithm [12, 13]. The parameters $\lambda$ and $h$ were set to 0.25 and 5 respectively, for all the experiments. These parameters were empirically optimized on three representative datasets to provide the best image quality and then the same parameters were employed in the remaining cases. A weight of 1.0 was employed for the Beltrami constant as indicated in Zosso and Bustin [11].

### Imaging

All imaging studies were performed on a 1.5 T MR scanner (Ingenia, Philips, Best, The Netherlands). T1 mapping was performed using the saturation-based 3D SASHA sequence described by Nordio et al. [7]. Phantom and cardiac studies used a 32-channel phased-array coil. The images from phantom and healthy subjects were collected from the scanner in DICOM format and were loaded into a computer for offline image processing and analysis using MATLAB (MathWorks, Natick, MA, USA).

### Phantom experiments

To evaluate the performance of the proposed 3D denoising algorithm with regard to the accuracy and precision of T1 values, a standardized phantom containing nine agar/NiCl_2_ vials was used, with T1 values ranging from 250 to 1500 ms [14]. The phantom was imaged using the gold-standard 2D inversion-recovery spin echo (IRSE), the 2D MOLLI (3-(3)-3-(3)-5) and the proposed 3D SASHA imaging techniques. Acquisitions parameters for the 2D IRSE sequence were: field of view (FOV) = 150 × 150 mm^2^, image resolution = 1.95 × 1.95 mm^2^, 10 mm slice thickness, 20 inversion times (TI) varying from 50 to 5000 ms, TR(i) = 7000 ms plus the variable inversion time TI(i), and TE = 6 ms. Acquisition parameters for the 2D MOLLI sequence included: FOV = 300 × 280 mm, image resolution = 1.7 mm × 2.1 mm, slice thickness = 10 mm, TR/TE = 2.6/1.3 ms, FA = 35°, acquired matrix = 176 × 132, scan time of 12 s. The acquisition parameters for the 3D SASHA sequence were: FOV = 300 × 300 × 90 mm, TR/TE = 3.2/1.6 ms, image resolution = 1.4 mm × 1.4 mm × 8 mm, FA = 35°, acquired matrix = 216 × 214, saturation time within the range of 100–700 ms for a heart rate of 60 beats per minute (bpm), parallel imaging with a SENSE factor of 2 in the phase-encoding direction, nominal scan time approximately 4 min. Nine T1-weighted images were acquired, including one image without any magnetization preparation and eight images at different saturation times. All three imaging techniques were reconstructed to the same in-plane resolution of 1.25 × 1.25 mm^2^.

### In vivo experiments

All subjects recruited to this study provided written informed consent, with study approval from the institutional review board (1/11/12). Fifteen healthy subjects with no history of cardiovascular disease underwent imaging using the 2D MOLLI and 3D SASHA sequences in the short-axis plane, using the same imaging parameters as for the phantom experiment. A total of 11 slices were acquired for the 3D SASHA sequence, with an oversampling factor of 1.14. The acquisition of the 3D SASHA sequence was performed in free-breathing with a nominal scan duration of 4:14 (min:s) for a heart rate of 60 bpm and 100% respiratory scan efficiency. A 1D diaphragmatic navigator was used for respiratory motion compensation, with a gating window of 5 mm and a tracking factor of 0.6 in the foot–head direction.

### Image analysis

The 3D Beltrami denoising was applied to the magnitude 3D T1-weighted images of the 3D SASHA acquisition prior to performing the T1 fitting. T1 maps were reconstructed offline using MATLAB with a three-parameter fitting model for the T1-weighted images before and after denoising [15]. A spatially matching region of interest (ROI) was manually drawn on the T1 map of the 2D IRSE (phantom only), 2D MOLLI, and 3D SASHA before (“non-denoised 3D SASHA”) and after (“denoised 3D SASHA”) denoising in all vials for the phantom experiment as well as in the septum of the myocardium for the in vivo data. The mean and standard deviation of the T1 measurements were calculated and used as an estimation of the accuracy and precision of the respective T1 mapping techniques. A Bland–Altman plot was used to compare the different imaging techniques for the phantom study. The precision measured on the 2D MOLLI and non-denoised and denoised 3D SASHA T1 maps of the healthy subjects were compared using a Mann–Whitney *U* test. An American Heart Association (AHA) segmentation [16] was used to compare the accuracy and precision of the 3D SASHA before and after denoising and for a 3D visualization of the left ventricle. The cardiac volume was represented in 16 segments and 3 slices (apex, mid and base), with the segment 17 corresponding to the blood pool. The value in each segment was averaged within the fifteen healthy subjects.

For statistical analysis, GraphPad Prism v5 for Windows (GraphPad Software, La Jolla, CA, USA) was used. Two-tailed values of *P* < 0.05 were considered statistically significant.

## Results

### Phantom experiments

**Fig. 1:**
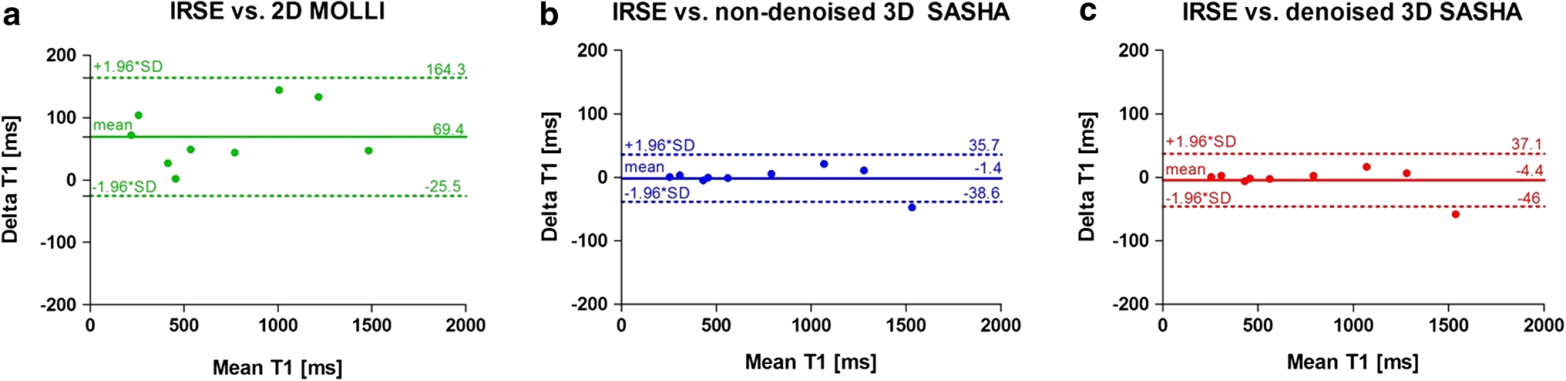
Bland–Altman plots comparing the gold-standard inversion recovery (IRSE) technique with the 2D MOLLI (**a**), the non-denoised 3D SASHA (**b**) and the denoised 3D SASHA (**c**) techniques acquired on the T1 phantom. The bias and 95% limits of agreement are reported for each graph

2D MOLLI and non-denoised and denoised 3D SASHA T1 maps are shown for the phantom experiment in supplementary Figure S1. Mean and standard deviation values obtained for all the vials in the phantom for 2D IRSE, 2D MOLLI and 3D SASHA before and after denoising are summarized in supplementary Table S2. The accuracy and precision of the T1 values obtained before and after applying the 3D denoising technique to the phantom images are shown in supplementary Figure S3. The standard deviation measured on the 3D SASHA before and after denoising is quite small (2–30 ms). As shown previously [7], 3D SASHA before denoising is highly accurate compared to the gold-standard 2D IRSE, while 2D MOLLI generally underestimates the T1 values. The Bland–Altman plots in Fig. 1 show the T1 underestimation typical of 2D MOLLI (Fig. 1a), and good agreement between the 2D IRSE and the 3D SASHA before (Fig. 1b) and after (Fig. 1c) denoising. For some vials of the phantom, the accuracy of the denoised 3D SASHA is slightly higher, with an increase of just a few milliseconds (about 0.3%).

### In vivo experiments

The total scan time for the 3D SASHA acquisition in healthy subjects was of 12:4 ± 1:1 (min:s), with an average scan efficiency of 36% and heart rates of 60–70 bpm.

**Fig. 2:**
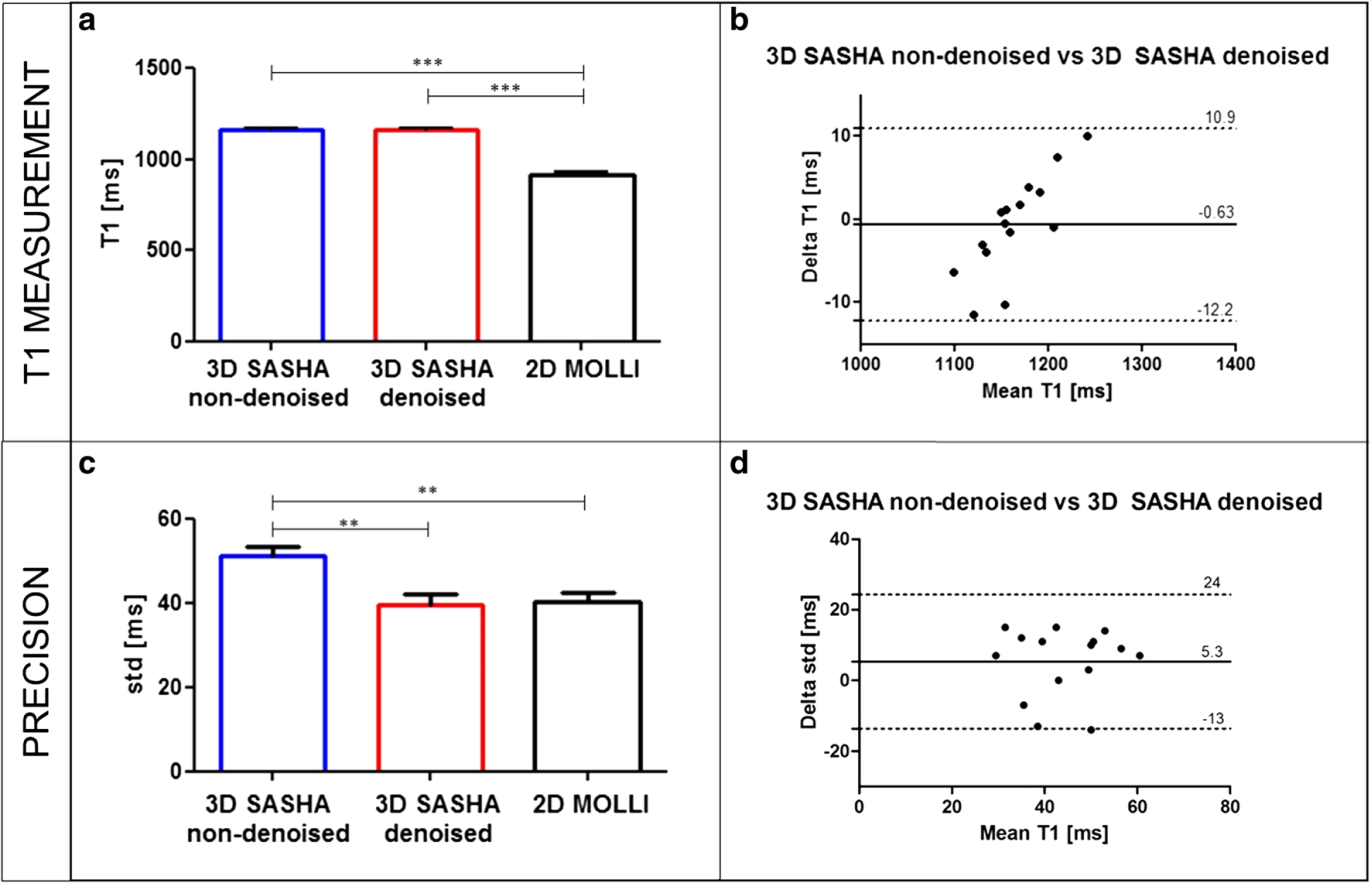
**a**–**c** Accuracy and precision of 2D MOLLI and 3D SASHA before (non-denoised 3D SASHA) and after (denoised 3D SASHA) 3D denoising averaged over the 15 healthy subjects. The precision was measured in a region of interest placed in the myocardial septum of a mid-ventricular slice. Results are expressed as mean ± standard deviation. Differences with statistical significance are identified by *P < 0.05. **b**–**d** Bland–Altman plot comparing the accuracy (**c**) and precision (**d**) of non-denoised 3D SASHA vs. denoised 3D SASHA. The bias and 95% limits of agreement are reported for each graph

Figure 2 shows the average accuracy and precision for all healthy subjects measured on the 2D MOLLI T1 maps and the non-denoised and denoised 3D SASHA T1 maps for a mid-ventricular slice (Fig. 2a–c), as well as the Bland–Altman plots comparing the T1 measurements and the precision of non-denoised 3D SASHA vs. denoised 3D SASHA (Fig. 2b–d). There was a significant difference between the accuracy measured with 2D MOLLI and the non-denoised and denoised 3D SASHA (*P *< 0.0001), which confirms the typical T1 underestimation of the 2D MOLLI technique (Fig. 2a). There was a statistical difference between the precision measured with 2D MOLLI and the non-denoised 3D SASHA (*P *= 0.005), and between the non-denoised and denoised 3D SASHA (*P* = 0.007). Conversely, there was no statistical difference between 2D MOLLI and the denoised 3D SASHA T1 maps (*P *= 0.95) (Fig. 2c).

The impact of the choice of the parameters *h* and λ on the image quality of the denoised 3D SASHA T1 map is shown in the supplementary Figure S4. The denoised T1 map becomes sharper with higher values for the parameter *h*. However, a value of *h* that is too high may result in patchy artifacts. The parameters $\lambda$ and $h$ were set to 0.25 and 5, respectively, for all the experiments.

**Fig. 3:**
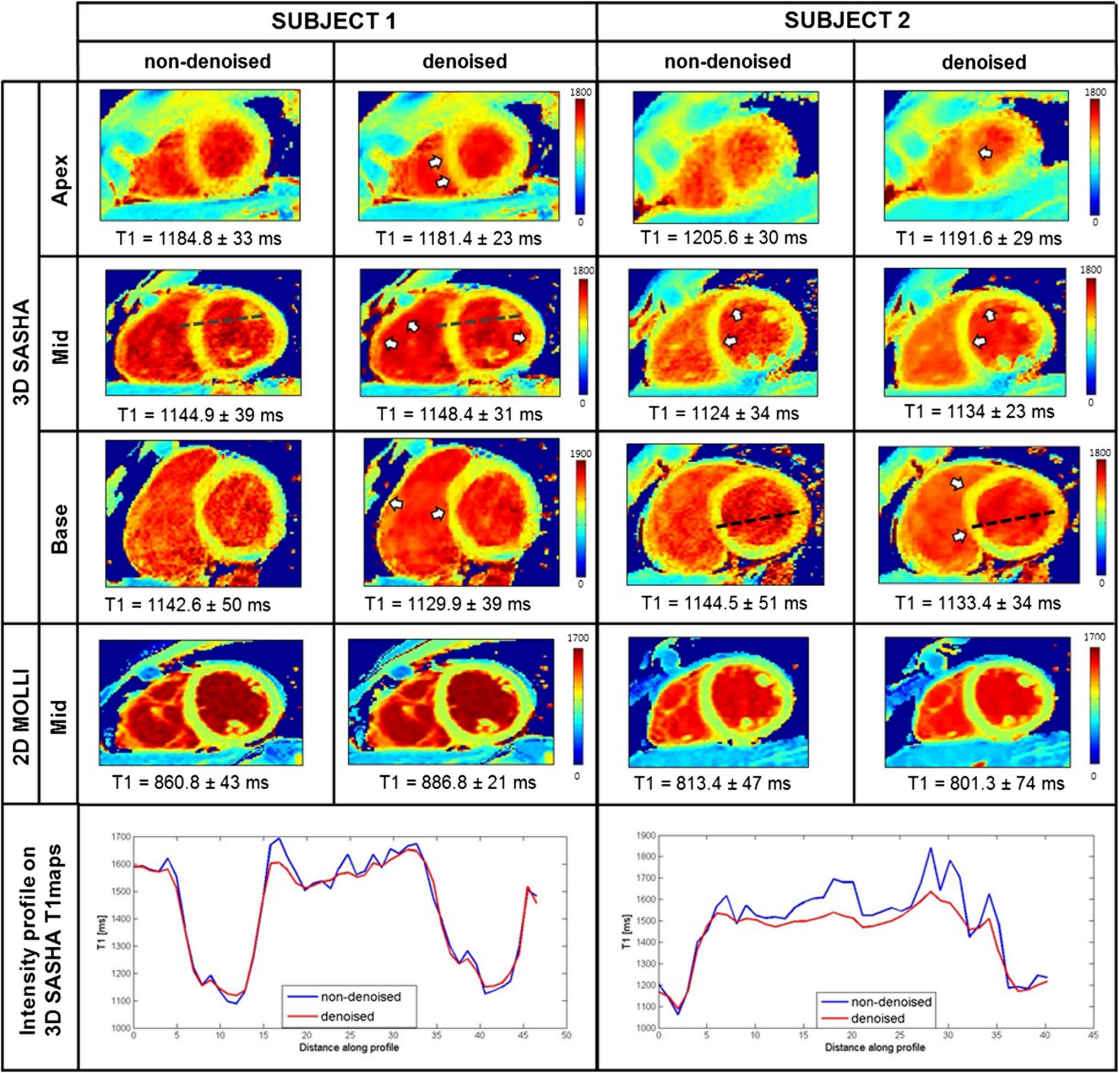
Myocardial T1 maps for two representative subjects. Representative T1 maps of the apex, mid and base slice before (non-denoised 3D SASHA) and after (denoised 3D SASHA) 3D denoising are shown in the first three rows. In the forth row, the 2D MOLLI T1 maps before and after denoising are shown. The intensity profiles in the last row show that after denoising, the delineation of the myocardial borders and the papillary muscles is maintained, while the signal in general is more homogeneous. T1 values are expressed as mean ± standard deviation

**Fig. 4:**
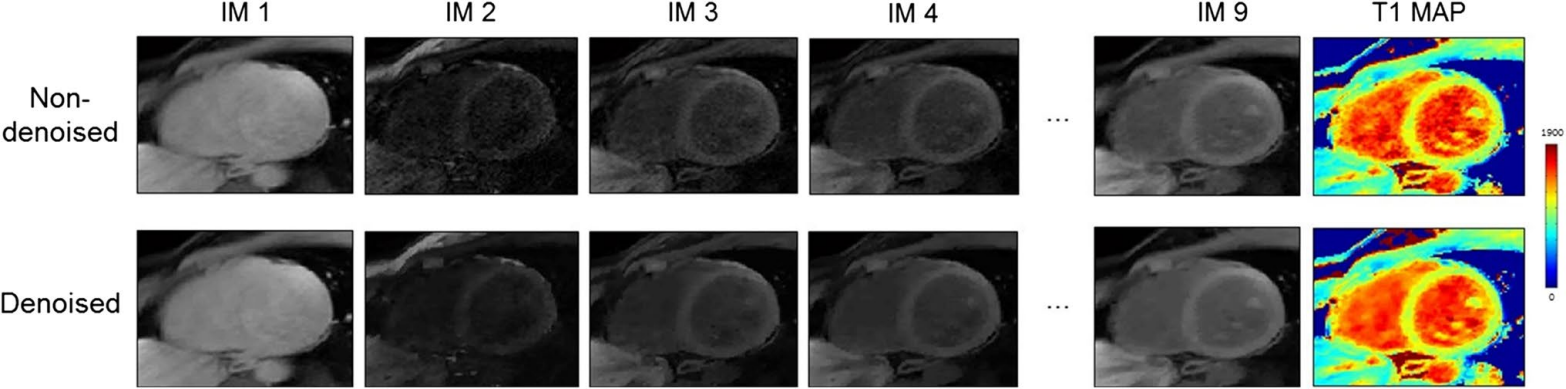
Single T1-weighted images before and after 3D Beltrami denoising and the corresponding 3D SASHA T1 map for a representative subject

The accuracy and precision measured for each healthy subject for the 3D SASHA T1 maps computed before and after denoising are shown in the supplementary Figure S5. The measurements were performed in an apical, mid-ventricular and basal septum. The accuracy was maintained after denoising, with no statistical difference among the three slices (apex: *P *= 0.38, mid: *P *= 0.88 and base: *P *= 0.35). There was a significant improvement in precision of measurements in all the three slices, respectively (apex: *P* = 0.013, mid: *P* = 0.007, base: *P* = 0.007). Figure 3 shows the 3D SASHA T1 maps before and after denoising for two representative subjects. The intensity profile in the bottom row of Fig. 3 shows that after denoising, the myocardial borders are maintained with 3D SASHA, while the signal is more homogeneous in general. The corresponding 2D MOLLI T1 maps before and after denoising are also included (mid-slice) in Fig. 3 for comparison purposes. The T1 values are slightly reduced along the inferolateral wall, which is probably due to susceptibility artifacts, residual motion or slice profile imperfection. Figure 4 shows the single T1-weighted images before and after 3D denoising and the corresponding 3D SASHA T1 map for a representative subject.

**Fig. 5:**
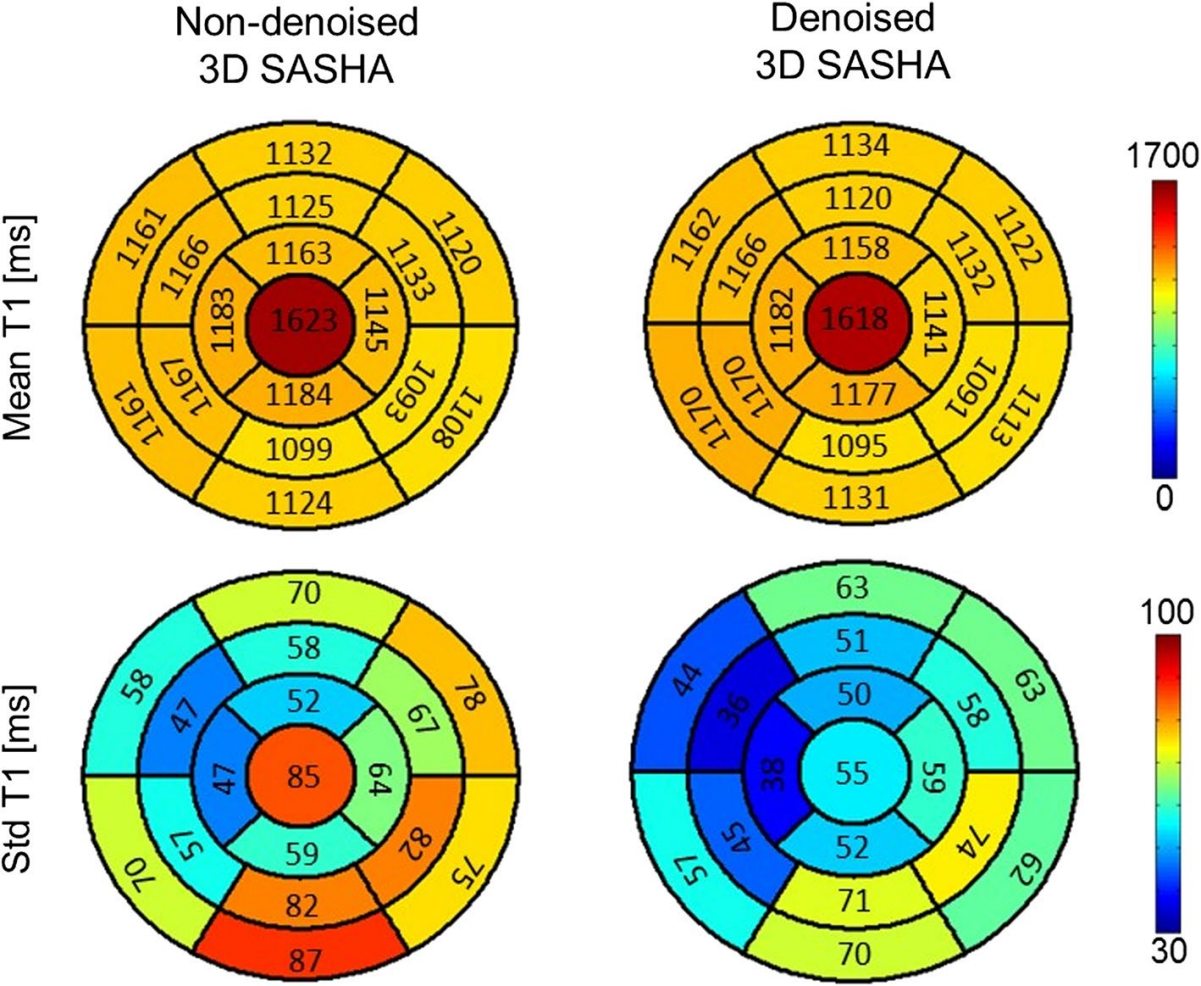
AHA segmentation of the left ventricle, shown for the non-denoised and denoised 3D SASHA (*n* = 15 subjects). The cardiac volume is represented in 16 segments and three slices (apex, mid and base), while the center represents the blood pool. The mean and standard deviation are indicated in all the segments. There is good homogeneity of the myocardial T1 values across the left ventricle, while the precision improves after denoising particularly for the blood pool. The precision is slightly lower in the inferolateral wall, probably due to susceptibility artifacts

Figure 5 shows the AHA segmentation of the non-denoised and denoised 3D SASHA approaches, for both the mean and standard deviation. In the whole volume the accuracy is maintained virtually constant between the non-denoised and denoised 3D SASHA approaches. The precision improves in all the segments after denoising, with a considerable improvement in precision for the blood pool represented in the central segment. There is a general increase of the standard deviation in the inferolateral wall, both before and after denoising, due to susceptibility artifacts or slice profile imperfection.

The processing time required for the Beltrami denoising on one 3D cardiac volume was about 1 min.

## Discussion

In this study, we introduced a 3D free-breathing SASHA T1 mapping framework with high accuracy and improved precision following implementation of a denoising step. 3D SASHA T1 mapping is characterized by higher spatial resolution and volumetric coverage and higher accuracy than the 2D MOLLI technique used clinically; however, its precision is lower [4, 7]. Here we have shown that it is feasible to obtain precision comparable to that of the 2D MOLLI technique while maintaining high accuracy and whole-heart coverage characteristic for 3D SASHA. This is achieved by applying a Beltrami denoising method to the T1-weighted 3D SASHA images prior to the T1 fitting. The denoising technique, proposed for 2D T1 mapping in Bustin et al. [8], imposes edge-preserving regularity and exploits the co-occurrence of 3D spatial gradients in the native images by incorporating a multi-contrast Beltrami regularization.

In contrast to the technique presented in Weingartner et al. [10], where multi-slice denoising using local neighborhood averaging for each pixel was performed, we utilized 3D spatial gradients to exploit the redundancy and sparsity of the whole 3D volume and Beltrami regularization to exploit sparsity in the T1 recovery dimension. This sparse formulation has the advantage of being robust to high levels of noise and maintaining sharp edges, while avoiding computationally expensive reconstruction often associated with local filters. Compared to the 2D denoising technique introduced in Bustin et al. [8], our framework further exploits redundancy and sparsity in the whole 3D volume and provides whole-heart volumetric coverage with high in-plane spatial resolution.

As demonstrated in the phantom experiment, the accuracy of the 3D SASHA T1 map before and after denoising is comparable to the gold-standard IRSE technique, with a slight increase of a few milliseconds for some vials. In terms of precision, the improvement is small, due to the small range of standard deviation. Conversely, with the 2D MOLLI imaging technique, the myocardial T1 was underestimated, as shown previously [2, 17]. In vivo experiments in healthy subjects demonstrated preserved accuracy of non-denoised and denoised 3D SASHA. In addition, the precision was considerably improved after denoising, with no statistical difference compared to 2D MOLLI T1 maps. The AHA segmentation allows for better visualization of the effect of the 3D denoising method on the whole volume, in terms of both accuracy and precision. The precision in general is improved across the whole left ventricle, as well as in the blood pool indicated in the central segment. In fact, the denoising should have a larger effect in areas with more noise or noise-like structures. As the blood pool signal is more inhomogeneous, we expect the denoising to be more efficient in that area. The variability in the precision measured in the myocardial inferolateral wall could be explained by the susceptibility artifacts and some residual motion [18]. After denoising, the delineation of the myocardial borders and papillary muscles is maintained, while the signal in general is more homogeneous. For respiratory motion compensation, the 3D SASHA imaging sequence is combined with a 1D diaphragmatic navigator gating and tracking. The navigator generally allows for respiratory scan efficiency of ~ 50%; however, markedly lower scan efficiency (20–30%) is often observed in patients with more irregular breathing patterns. An alternative respiratory motion compensation technique, such as image-based navigation or self-navigation [19, 20], would permit 100% respiratory scan efficiency, thus reducing the scan time and consequently potential additional bulk motion artifacts associated with lengthy scans. Compared to 3D SASHA, 2D MOLLI permits the acquisition of one slice in only 12 s of breath-hold. However if several slices are acquired, resting periods of at least the same duration as the breath-hold are necessary between the acquisition of each slice. Consequently, to acquire several slices and to achieve the same volumetric coverage of the 3D SASHA presented here, 2D MOLLI would require a total scan time of about 4 min. In addition, 2D MOLLI has lower image resolution than 3D SASHA; a higher resolution would require a longer acquisition window, making the MOLLI scan more susceptible to cardiac motion.

The denoising approach applied in this study is a post-processing step and thus comes at no additional acquisition or reconstruction cost. The additional post-processing time of ~ 1 min per 3D volume to execute the denoising technique is reasonable and, if clinically validated, inline implementation would be feasible. The integration of the denoising algorithm in the reconstruction step may accelerate and simplify the whole framework and will be investigated in future work. Overall, 3D SASHA with denoising appears to be a promising alternative for providing accurate T1 values while reducing variability, thus addressing current barriers to the wide-scale adoption of quantitative myocardial tissue characterization techniques in the clinical setting.

## Conclusion

We have demonstrated the feasibility of the 3D Beltrami denoising technique to preserve accuracy and improve precision in myocardial 3D SASHA T1 mapping. Ultimately, this technique could enable accurate and more precise reconstruction of myocardial T1 maps, with the potential to offer better visual image analysis and improved performance of post-processing procedures such as image registration and segmentation.

## Electronic supplementary material

Below is the link to the electronic supplementary material.

Figure S1: T1 maps of the T1 phantom using the following imaging sequences: IRSE, 2D MOLLI, 3D SASHA before and after denoising (TIFF 850 kb)

Figure S2: Mean and standard deviation measured in all the vials of the T1 phantom using the imaging sequences: IRSE, 2D MOLLI, 3D SASHA before (non-denoised) and after (denoised) denoising (TIFF 274 kb)

Figure S3: Comparison of the accuracy and precision of the 3D SASHA T1 map measured before (non-denoised 3D SASHA) and after (denoised 3D SASHA) applying the 3D denoising technique on all the vials of the phantom. Each color in the graph corresponds to a different vial of the T1 phantom (TIFF 163 kb)

Figure S4: Impact of the choice of the parameters *h* and λ on the image quality of the denoised 3D SASHA T1 map. The sharpness of the structures in the T1 map is controlled by the parameter h, while the denoising effect is controlled by the parameter λ. The higher the sharpness parameter h, the sharper the denoised T1 map. However, a value of *h* that is too high results in patchy artifacts (TIFF 1417 kb)

Figure S5: Comparison of the accuracy and precision of the 3D SASHA T1 map measured before (non-denoised 3D SASHA) and after (denoised 3D SASHA) applying the 3D denoising technique. The values have been measured in the septum of the myocardium for all subjects in the apical, mid-ventricular and base slices. Each color in the graph corresponds to a different healthy subject (TIFF 604 kb)
