# Correction to: 3D SASHA myocardial T1 mapping with high accuracy and improved precision

**DOI:** 10.1007/s10334-018-0706-8

**Published:** 2018-10-08

**Authors:** Giovanna Nordio, Aurélien Bustin, Markus Henningsson, Imran Rashid, Amedeo Chiribiri, Tevfik Ismail, Freddy Odille, Claudia Prieto, René Michael Botnar

**Affiliations:** 1 School of Biomedical Engineering and Imaging Sciences, King’s College London, 4th Floor, Lambeth Wing, St Thomas’ Hospital, London, SE1 7EH UK; 2 Escuela de Ingeniería, Pontificia Universidad Católica de Chile, Santiago, Chile; 3 Imagerie Adaptive Diagnostique et Interventionnelle, INSERM et Université de Lorraine, Nancy, France; 4 CIC-IT 1433, INSERM, Université de Lorraine, CHRU de Nancy, Nancy, France

## Correction to: Magnetic Resonance Materials in Physics, Biology and Medicine 10.1007/s10334-018-0703-y

The original version of this article unfortunately contained a mistake. The presentation of Equation was incorrect. The corrected equation is given below.

The original article has been corrected.

$$ \hat{m}_{i} = {\text{argmin}}_{{m_{i} }} \left\{ {\left\| {m_{i} - d_{i} } \right\|_{2}^{2} + \lambda \sqrt {1 + \beta^{2} \mathop \sum \limits_{i = 1}^{n} \left| {\nabla^{w} m_{i} } \right|^{2} } } \right\} $$

