# Supplementary figures and images for: 3D SASHA myocardial T1 mapping with high accuracy and improved precision
Source: MAGMA. 2018 Sep 6;32(2):281–9. doi: 10.1007/s10334-018-0703-y (PMC6424941; doi:10.1007/s10334-018-0703-y)

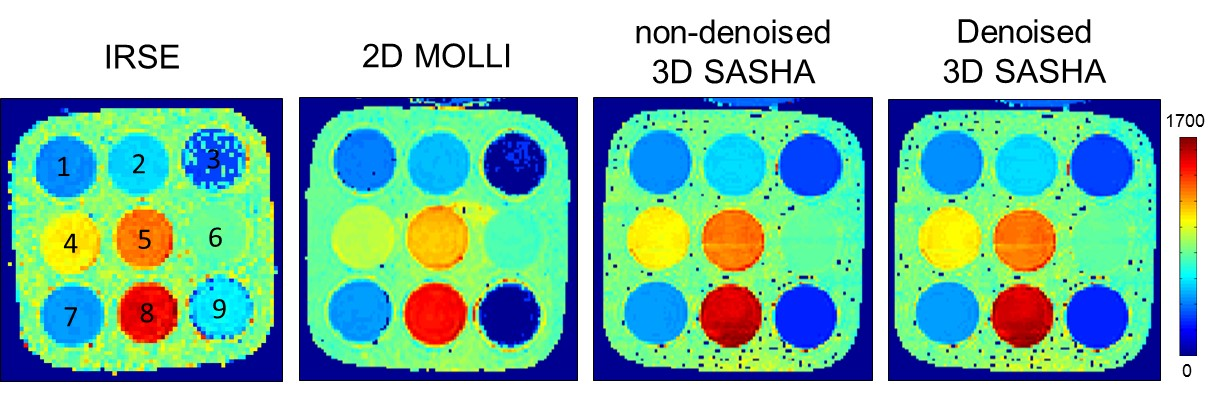

Supplement: Supplementary file 1 — Figure S1: T1 maps of the T1 phantom using the following imaging sequences: IRSE, 2D MOLLI, 3D SASHA before and after denoising (TIFF 850 kb) [file 10334_2018_703_MOESM1_ESM.tif]

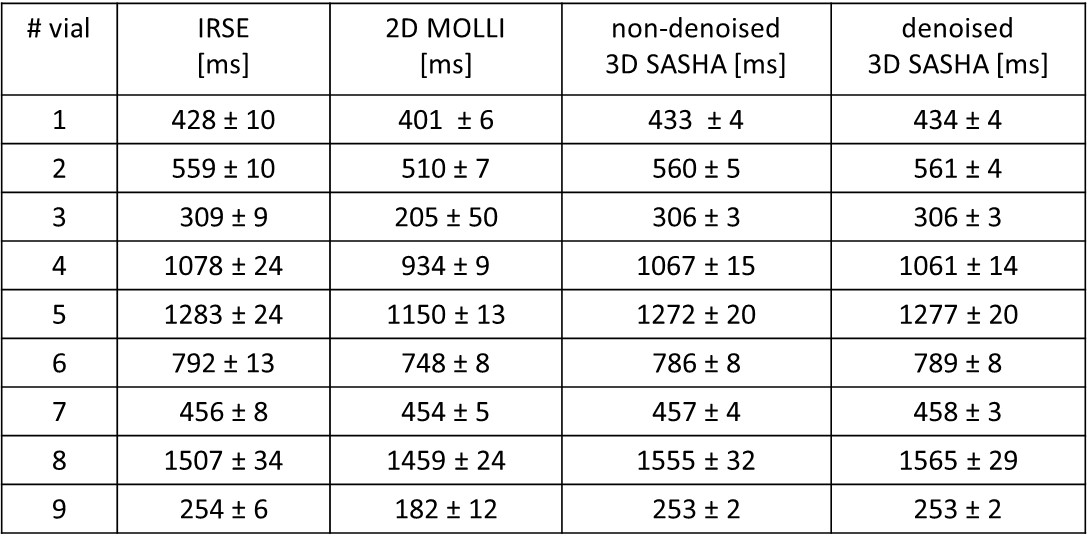

Supplement: Supplementary file 2 — Figure S2: Mean and standard deviation measured in all the vials of the T1 phantom using the imaging sequences: IRSE, 2D MOLLI, 3D SASHA before (non-denoised) and after (denoised) denoising (TIFF 274 kb) [file 10334_2018_703_MOESM2_ESM.tif]

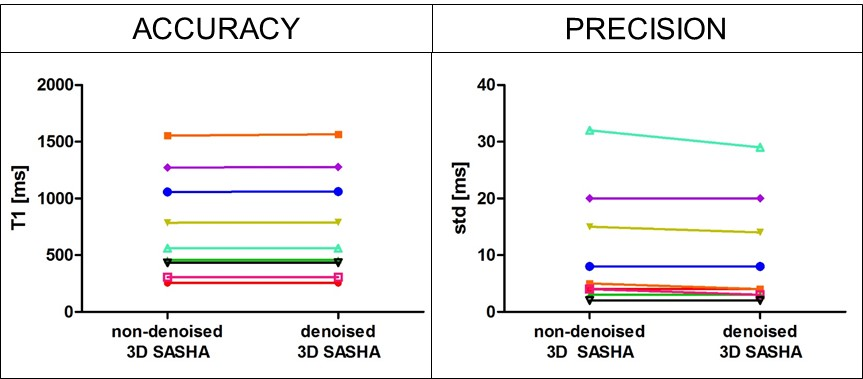

Supplement: Supplementary file 3 — Figure S3: Comparison of the accuracy and precision of the 3D SASHA T1 map measured before (non-denoised 3D SASHA) and after (denoised 3D SASHA) applying the 3D denoising technique on all the vials of the phantom. Each color in the graph corresponds to a different vial of the T1 phantom (TIFF 163 kb) [file 10334_2018_703_MOESM3_ESM.tif]

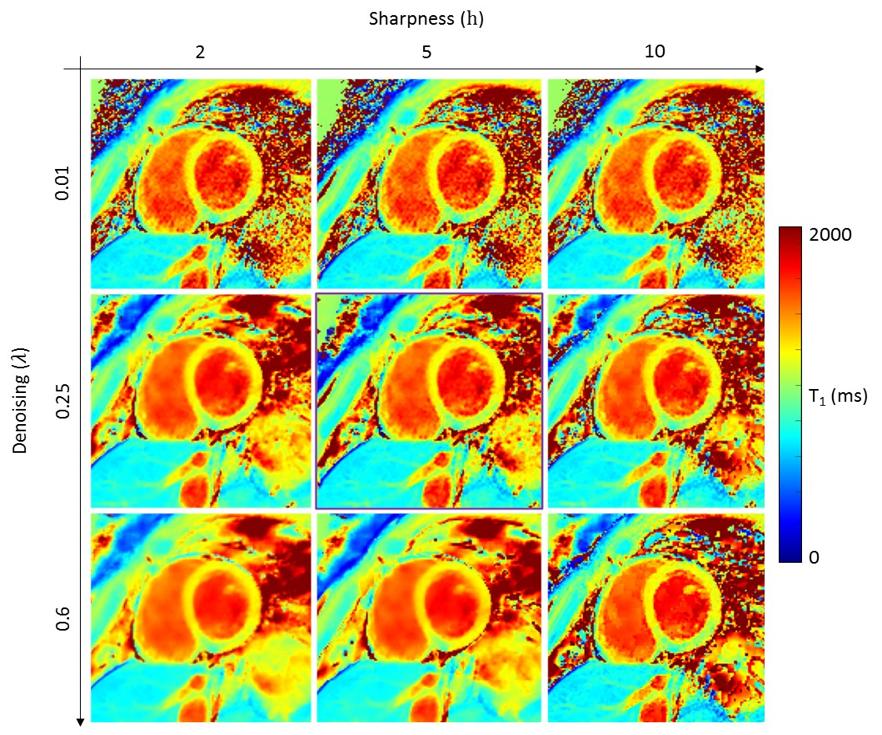

Supplement: Supplementary file 4 — Figure S4: Impact of the choice of the parameters h and λ on the image quality of the denoised 3D SASHA T1 map. The sharpness of the structures in the T1 map is controlled by the parameter h, while the denoising effect is controlled by the parameter λ. The higher the sharpness parameter h, the sharper the denoised T1 map. However, a value of h that is too high results in patchy artifacts (TIFF 1417 kb) [file 10334_2018_703_MOESM4_ESM.tif]

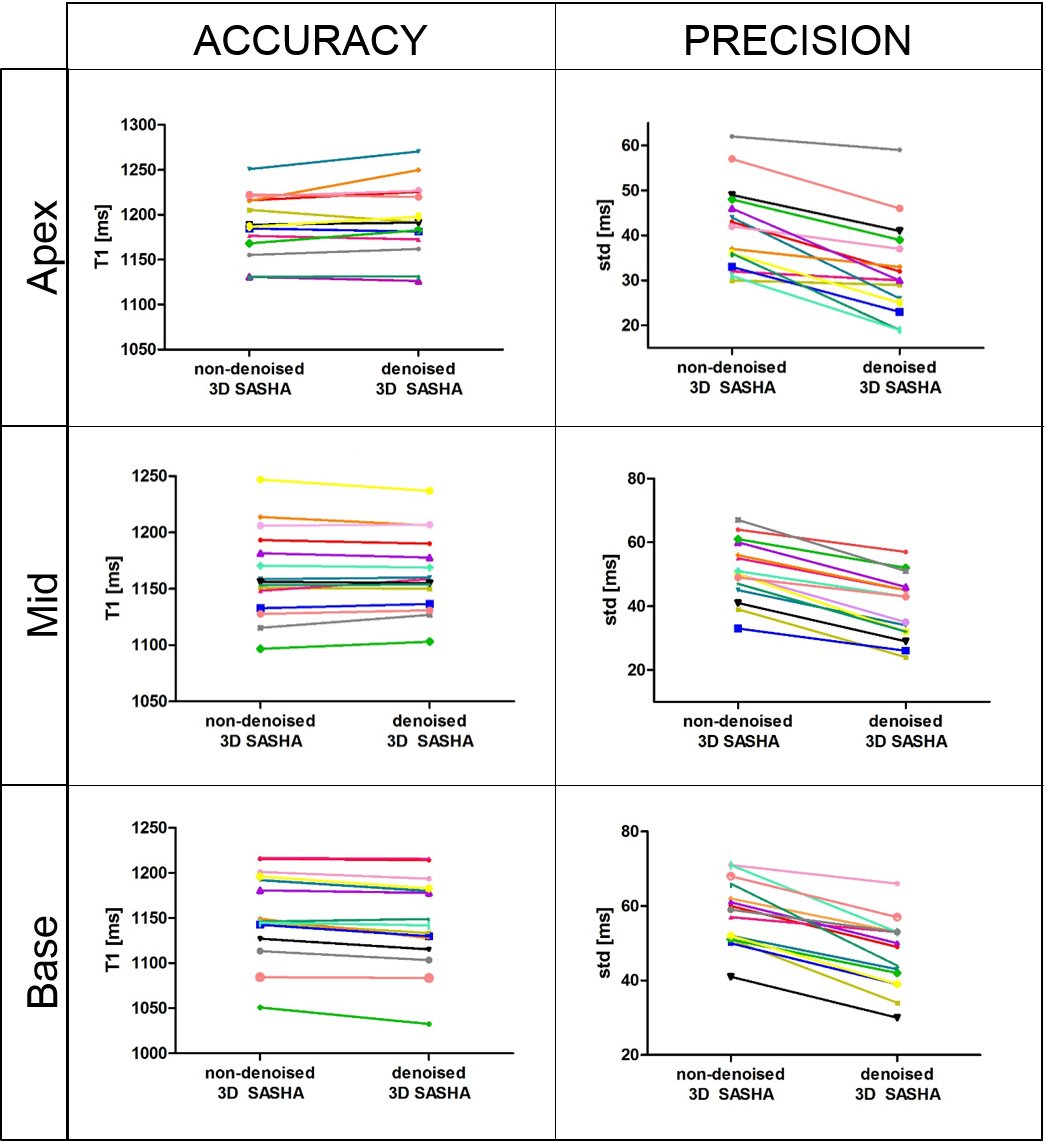

Supplement: Supplementary file 5 — Figure S5: Comparison of the accuracy and precision of the 3D SASHA T1 map measured before (non-denoised 3D SASHA) and after (denoised 3D SASHA) applying the 3D denoising technique. The values have been measured in the septum of the myocardium for all subjects in the apical, mid-ventricular and base slices. Each color in the graph corresponds to a different healthy subject (TIFF 604 kb) [file 10334_2018_703_MOESM5_ESM.tif]
